# Real-Time Evaluation of Anterior Choroidal Artery Patency During Aneurysm Clipping

**DOI:** 10.7759/cureus.495

**Published:** 2016-02-14

**Authors:** Daniel Felbaum, David Y Zhao, Vikram V Nayar, Christopher G Kalhorn, Kevin M McGrail, Allen S Mandir, Robert E Minahan

**Affiliations:** 1 Neurosurgery, Medstar Georgetown University Hospital; 2 Neurology, Medstar Georgetown University Hospital

**Keywords:** aneurysm, anterior choroidal aneurysm, intraoperative monitoring, motor evoked potentials

## Abstract

Inadvertent occlusion of the anterior choroidal artery during aneurysm clipping can cause a disabling stroke in minutes. We evaluate the clinical utility of direct cortical motor evoked potential (MEP) monitoring during aneurysm clipping, as a real-time assessment of arterial patency, prior to performing indocyanine green videoangiography.

Direct cortical MEPs were recorded in seven patients undergoing surgery for aneurysms that involved or abutted the anterior choroidal artery. The aneurysms clipped in those seven patients included four anterior choroidal artery aneurysms and six posterior communicating artery aneurysms. Serial MEP recordings were performed during the intradural dissection, aneurysm exposure, and clip placement. A significant change in MEPs after clip placement would prompt immediate inspection and removal or repositioning of the clip. If the clip placement appeared satisfactory and MEP recordings were stable, then an intraoperative indocyanine green videoangiogram was performed to confirm obliteration of the aneurysm and patency of the arteries.

Seven patients underwent successful clipping of anterior choroidal artery aneurysms and posterior communicating artery aneurysms using direct cortical MEP monitoring, with good clinical and radiographic outcomes. In six patients, no changes in MEP amplitudes were observed following permanent clip placement. In one patient, a profound decrease in MEP amplitude occurred 220 seconds after placement of a permanent clip on a large posterior communicating aneurysm. An inspection revealed that the anterior choroidal artery was kinked. The clip was immediately removed, and the MEP signals returned to baseline shortly thereafter. A clip was then optimally placed, and the patient awoke without neurologic deficit.

Direct cortical MEPs are a useful adjunct to standard electrophysiologic monitoring in aneurysm surgery, particularly when the anterior choroidal artery or lenticulostriate arteries are at risk. When these arteries are occluded, infarction may occur before the occlusion is detected by indocyanine green videoangiography or intraoperative angiography. The use of MEPs allows real-time detection of ischemia to subcortical motor pathways.

## Introduction

Intraoperative monitoring of motor evoked potentials (MEPs) may increase the safety of aneurysm surgery, particularly when there is a significant risk of anterior choroidal artery occlusion. Focal subcortical ischemia affecting the pyramidal tracts would generally be undetected by monitoring of somatosensory evoked potentials (SSEPs) or the electroencephalogram.

Despite the potential advantages of MEP monitoring, the technique has not garnered widespread use in aneurysm surgery. There is a paucity of literature describing direct cortical stimulation during aneurysm surgery as a way to monitor MEPs without causing the hazardous patient movement that might occur with transcranial stimulation [1-3]. To our knowledge, the combined use of direct cortical motor evoked potentials and indocyanine green (ICG) videoangiography has not been previously described for aneurysm surgery.

We describe our recent experience with the use of direct cortical stimulation for MEP monitoring during the microsurgical clipping of aneurysms involving the communicating segment of the internal carotid artery. The technique provided real-time feedback about anterior choroidal artery patency prior to assessment with ICG videoangiography. We illustrate how this feedback allowed timely repositioning of an aneurysm clip, and we discuss nuances of the technique. 

## Technical report

### Patient population

After written informed consent, seven patients underwent microsurgical clipping of aneurysms involving the communicating segment of the internal carotid artery. The average patient age was 66.5 years (range: 55-81 years). Two patients had presented with subarachnoid hemorrhage; the remaining patients were diagnosed with unruptured intracranial aneurysms. Of the aneurysms clipped, four were anterior choroidal artery aneurysms, and six were posterior communicating artery aneurysms. In two patients, both a posterior communicating artery aneurysm and an anterior choroidal artery aneurysm were clipped on the same side. One patient underwent staged bilateral craniotomies for clipping of aneurysms. In all cases, MEP monitoring with direct cortical stimulation was performed to identify potential occlusion of the anterior choroidal artery during aneurysm exposure and clipping. All patients subsequently underwent ICG videoangiography to confirm obliteration of the aneurysm and patency of the involved arteries.

### Anesthesia

General anesthesia was induced and maintained with a bolus and continuous infusion of propofol, remifentanil, and vecuronium. When inhalational gases were used, they were kept at consistent levels with the highest concentrations approaching 0.5 MAC (minimum alveolar concentration). The level of neuromuscular blockade was also monitored in order to interpret MEP results. Train-of-four analyses were performed following 2 Hz stimulation of the ulnar nerves at the wrist to the thenar muscles and of the posterior tibial nerves at the ankle to the  abductor hallucis muscles. Consistency in neuromuscular blockade was maintained during the procedure with any changes addressed by the anesthesia team adjusting the vecuronium drip rate. Some level of neuromuscular blockade was maintained in all of the patients to reduce the risk of spontaneous movement or potential induced movement from MEP testing.

MEP and SSEP stimulation and acquisition were performed using portable XLtek EPWorks stations (Natus Neurology, Middleton, WI). All subcutaneous recording and stimulation needle electrodes were inserted during the time of initial anesthesia and surgical positioning.  

SSEPs were recorded at peripheral points (Erb’s point and popliteal fossa) at the neck, and from cortical leads following stimulation at the wrist and at the posterior tibial nerves. MEPs were recorded from subcutaneous needle electrodes at bilateral deltoid, thenar, tibialis anterior, gastrocnemius, and abductor hallucis muscles. Additional muscles were sometimes used contralateral to the surgical site at the biceps, triceps, and/or orbicularis oris muscles. In order to idealize the location and consistency of direct cortical stimulation, the primary motor gyrus was first localized using phase-inversion identification following ulnar nerve stimulation. An 8-contact subdural linear grid electrode (Ad-Tech Medical Instrument Corp., Racine, WI) was placed directly on the cortical surface orthogonal to the best identification of the central sulcus. Each grid point was referenced to Fz and the eight traces analyzed for phase inversion [4]. Once a satisfactory position over the primary motor cortex was obtained, the leads to the grid were switched from recording inputs to stimulation inputs. Multiple spots on the grid were used to identify the best MEPs. Low-intensity stimulation (~15 – 30 mA; 5 pulses; 375 Hz rate; 0.5 msec duration) was delivered through one of the grid contacts as an anode and a needle electrode at the first dorsal interosseous (FDI) muscle as the cathode. Typically, one or two grid contacts were used as MEPs sites and the results followed in contralateral muscles. MEPs were successfully obtained in each of the patients. The anesthetic environment was maintained as stable as allowable, and changes not explained by anesthetics were reported to the surgeons within one to two minutes.  

A modified longitudinal bipolar montage was used for continuous EEG recording, such that four channels of the EEG were recorded for each hemisphere. Frontal, temporal, central, and occipital electrodes were placed with adjustments as necessary to avoid the surgical incision.

### Operative technique

**Figure 1:**
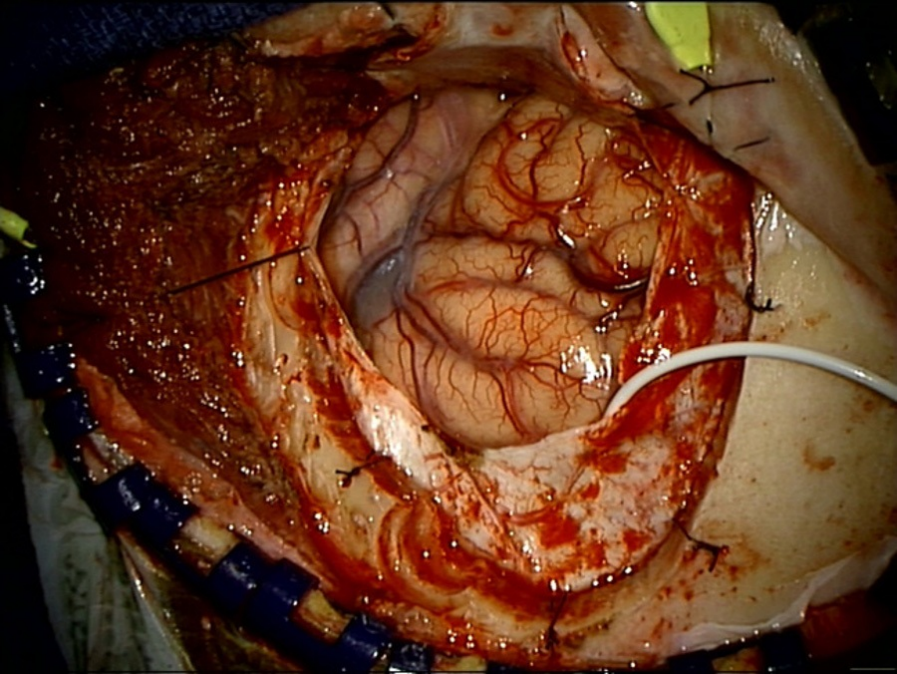
Placement of subdural grid A left pterional craniotomy and dural opening have been performed. A 1 x 8 electrode strip has been passed into the subdural space under the posterior edge of the dural opening, with electrodes overlying the precentral and postcentral gyri.

Under general anesthesia, and with the patient’s head in three-point pin fixation, monitoring of SSEPs and EEGs was performed by a neurologist throughout the surgery. After a pterional craniotomy was performed and the dura was opened, a 1 x 8 electrode grid was passed into the subdural space posterior to the dural opening, with concurrent gentle irrigation. The grid was positioned so that electrodes would overlay the expected location of hand representation on both the precentral and postcentral gyri. Medial passage of the grid was avoided, so as not to avulse bridging veins in the subdural space. The electrode cable was positioned so that it would not impede the microsurgical approach to the aneurysm (Figure 1).

As described above, electrodes overlying the primary motor cortex were identified by SSEP phase reversal. Direct cortical stimulation was then performed for MEP recording. If stimulation of proximal upper extremity muscle groups created noticeable patient movement, the electrode grid was repositioned laterally to avoid stimulating more axial or proximal muscles. The position of the electrode grid was optimized prior to microsurgical dissection near the aneurysm to allow for MEP monitoring without causing any motion noticeable to the surgeon.

Throughout the intradural portion of the procedure, MEP recordings were repeatedly performed along with the routine electrophysiologic monitoring of SSEPs and EEG. Microsurgical dissection and clipping of the aneurysm was performed, using brief temporary occlusion of the parent artery when required. Immediately after clip placement, MEPs were performed to assess patency of the anterior choroidal artery. Once clip placement appeared satisfactory, both by visual inspection and by electrophysiologic monitoring, indocyanine green (ICG) videoangiography was performed as previously described by Raabe, et al. [5]. Briefly, a 25 mg bolus of ICG dye solution in 10 ml of saline is injected intravenously. Simultaneously, the operative field is illuminated using the Zeiss OPMI Pentero (Carl Zeiss Meditec, Dublin, CA) and a near-infrared-sensitive camera documents the pattern of blood flow.

### Case illustration

**Figure 2:**
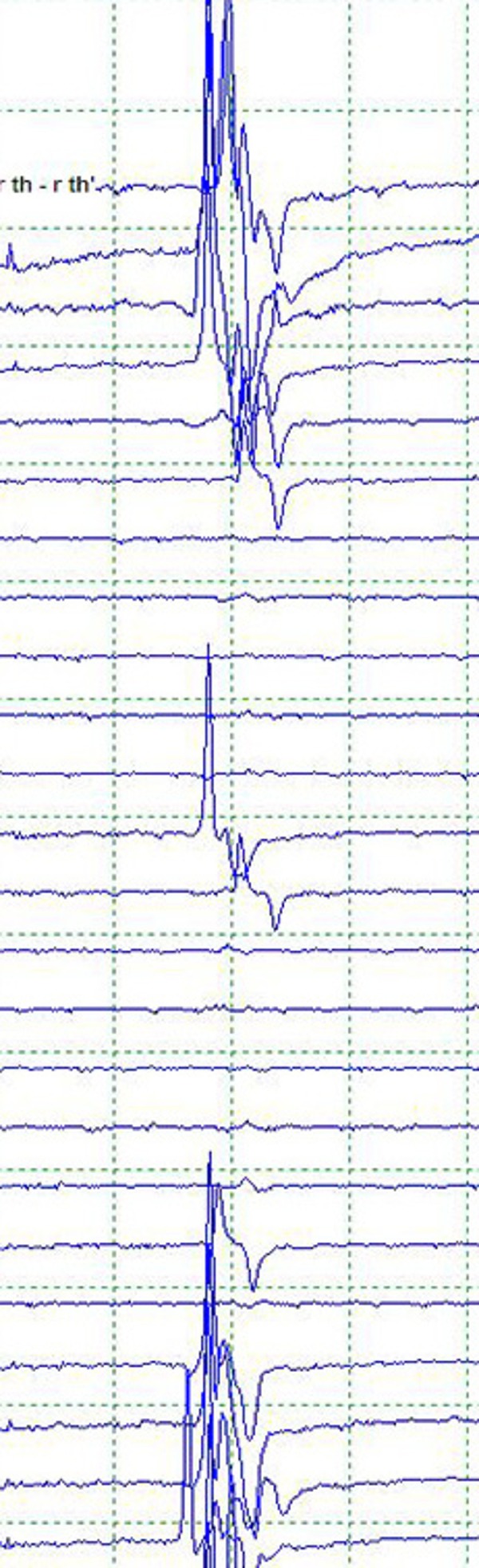
Compound motor action potential prior to clip placement Stack of contralateral thenar CMAPs following direct cortical stimulation prior to clip placement, during, and after replacement to show a return of responses.

A 68-year-old female underwent a left pterional craniotomy for clipping of two unruptured aneurysms: a 7 x 5 mm wide-necked posterior communicating artery aneurysm (Pcom) that projected posteromedially and a 3 x 2 mm anterior choroidal artery aneurysm. A neurologist performed SSEP and EEG monitoring throughout the surgery and MEP monitoring with direct cortical stimulation during the intradural portion of the case. After dissection of the anterior choroidal artery from the large Pcom aneurysm, this aneurysm was initially occluded with a 7.5 mm right-angled fenestrated clip. By visual inspection, all arteries appeared preserved, and the aneurysm obliterated. However, a profound decrease in MEP amplitude was noted 220 seconds after the clip was applied (Figure 2).

**Figure 3:**
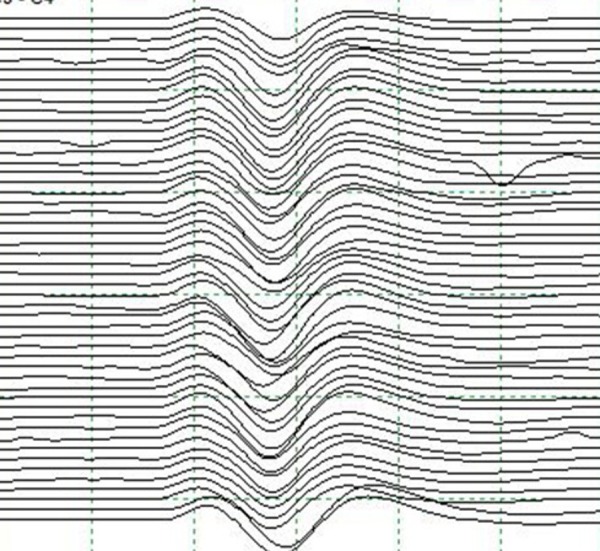
Somatosensory evoked potentials during clip placement Consistent SSEPs following contralateral median nerve stimulation during initial clip placement.

There were no changes in SSEP (Figure 3) or EEG monitoring at this time. Re-inspection suggested that the anterior choroidal artery was kinked, causing occlusion. The clip was removed, and MEPs returned to baseline. The Pcom aneurysm was then obliterated with two 5 mm right-angled fenestrated clips, and the anterior choroidal artery aneurysm with a 4.5 mm curved clip, without any changes to MEPs, SSEPs, or EEG. After satisfactory electrophysiologic signals and visual inspection, aneurysm occlusion with vessel preservation was confirmed by ICG videoangiography. The patient awoke after surgery without neurologic deficit, and no infarct was seen on postoperative CT.

## Discussion

The intraoperative electrodiagnostics that are most commonly used during intracranial aneurysm surgery are somatosensory evoked potentials (SSEPs) and the electroencephalogram (EEG). However, neither of these modalities would alert the surgeon to focal subcortical ischemia affecting the pyramidal tracts that can occur with anterior choroidal artery occlusion or lenticulostriate artery occlusion [6].

There have been several studies describing the use of transcranial motor evoked potentials in aneurysm surgery [3, 7-10]. Collectively, the studies demonstrate the value of MEPs in predicting and potentially preventing postoperative motor deficits. Neuloh and Schramm demonstrated the superiority of MEP monitoring over microvascular Doppler ultrasonography in detecting motor impairment from subcortical ischemia [11]. Nevertheless, motor evoked potential (MEP) monitoring remains unpopular among aneurysm surgeons, primarily because of the concern for hazardous patient movement during the delicate dissection around an aneurysm. 

Unlike transcranial stimulation, direct cortical stimulation for MEPs utilizes a much lower stimulation intensity to produce a response. Direct cortical stimulation provides focal muscle activation and can be designed to produce less patient movement. There are very few studies describing direct cortical stimulation for MEP monitoring in aneurysm surgery [1-3]. These reports suggest that MEPs elicited by direct cortical stimulation are a safe and reliable method for detecting ischemia in the subcortical motor pathways.

Despite the potential utility of direct cortical stimulation for MEPs, the technique has not become routine in aneurysm surgery. In the current era, aneurysm surgeons may rely on ICG videoangiography to confirm patency of the anterior choroidal artery or lenticulostriate arteries. In our experience, there is typically a delay of several minutes, from the time a clip is first applied to the time an ICG videoangiogram is completed. That delay may result from the placement of additional clips, readjustment of clips, visual inspection of the field, or preparation for the videoangiogram. During that period, infarction of subcortical motor pathways supplied by end arteries, like the anterior choroidal artery and lenticulostriate arteries, could occur. 

In our small series of patients, we have employed direct cortical MEPs to provide real-time feedback of anterior choroidal artery patency during aneurysm clipping, prior to performing an intraoperative ICG videoangiogram. To our knowledge, this is one of the first reports describing the use of both direct cortical MEP monitoring and ICG videoangiography in aneurysm surgery. We illustrate a case in which MEPs proved particularly useful in the early identification and correction of anterior choroidal artery occlusion.

## Conclusions

While ICG videoangiography has demonstrated efficacy in verifying anterior choroidal artery patency after aneurysm clipping, this imaging study is often delayed for several minutes, during which time infarction of the subcortical motor pathways could occur. Motor evoked potential monitoring with direct cortical stimulation can provide real-time assessment of anterior choroidal artery patency, allowing early clip readjustment when necessary. 
